# Repression of the Antioxidant Pyrroloquinoline Quinone in Skin Aging Induced by Bmi-1 Deficiency

**DOI:** 10.1155/2022/1732438

**Published:** 2022-02-09

**Authors:** Jing Li, Musang Liu, Shuo Liang, Yue Yu, Mufeng Gu

**Affiliations:** ^1^Institute of Dermatology, Chinese Academy of Medical Science and Peking Union Medical College, Nanjing, 210042 Jiangsu, China; ^2^Department of Anatomy, Key Laboratory for Aging and Disease, Nanjing Medical University, Nanjing, 211166 Jiangsu, China; ^3^Department of Dermatology, The Affiliated Suqian First People's Hospital of Nanjing Medical University, Suqian, 223800 Jiangsu, China

## Abstract

It is uncertain whether Bmi-1 deficiency could lead to skin aging by redox imbalance and DNA damage. In this study, we first confirmed that Bmi-1 had a relatively high expression level in the skin and Bmi-1 expression levels gradually decreased with age. Then, we studied the role of Bmi-1 in the skin using a Bmi-1^−/−^ mouse model. Bmi-1^−/−^ mice were supplemented with or without pyrroloquinoline quinone (PQQ) for 5 weeks, and their skin phenotypes were compared with Bmi1^−/−^ and wild-type littermates. Our results showed that Bmi-1^−/−^ mice displayed decreased vertical thickness of skin, sparse hair follicles, and thinner and more irregular collagen bundles. Mechanistically, increased oxidative stress with reducing antioxidant capacity and induced DNA damage occurred in Bmi-1^−/−^ mice. Subsequently, this would lead to reduced cell proliferation, increased cell senescence and matrix metalloproteinases (MMPs), and the degradation of fibroblast function and further reduce collagen synthesis. All pathological alterations in the skin of Bmi-1^−/−^ mice were alleviated by PQQ supplementation. These results demonstrated that Bmi-1 might play a key role in protection from skin aging by maintaining redox balance and inhibiting DNA damage response and will be a novel and potential target for preventing skin aging.

## 1. Introduction

Skin aging is a complicated process which occurs under the influence of external factors (such as sunlight exposure, air pollution, smoking, alcohol, and malnutrition) or internal factors (such as age and genetic background). External aging is characterized by appearance of atrophy, actinic keratoses, hyperpigmentation, and wrinkles. Intrinsically aged skin appears to be loose with loss of elastic and collagen fiber networks, thinning skin and deepening of expression lines [1]. An in-depth study on skin changes associated with aging will be beneficial to improve skin appearance and promote optimal beauty.

Reports focusing on molecular mechanisms of skin aging showed that oxidative stress in skin is one of the core mechanisms mediating skin aging, which is true for intrinsic aging and even more for extrinsic aging [2, 3]. Intracellular and extracellular oxidative stress initiated by reactive oxygen species (ROS) accumulation can cause damage to lipid, protein, nucleic acid, and organelle, which advances to the occurrence of cell senescence. Senescent cells can result in increased secretion of cytokines, such as interleukin- (IL-) 1*α*, IL-6, growth factors, matrix metalloproteinases (MMPs), and tumor necrosis factor- (TNF-) *α* [4]. MMPs are an important kind of proteolytic enzyme which contributes to extracellular matrix (ECM) degradation and the destruction of collagen fibers in the skin [5]. Supplements with some antioxidants have been certified to be effective in enhancing resistance to oxidative stress and preventing skin aging [6]. Exogenous skin pretreatment with antioxidant-based lotions can protect skin from oxidative damage [7, 8]. Therefore, strategies that can ameliorate oxidative stress-induced senescence have been expected to be a therapeutic target for the prevention of skin aging, aiming to hold the promise of promoting skin beauty.

Lymphoma Mo-MLV insertion region 1 (Bmi-1) was initially identified as an oncogene, which is a member of the polycomb family of transcriptional repressors [9]. Bmi-1 is widely expressed in many tissues and proved to be essential for normal tissue function including the brain, bone, kidney, and hematopoiesis [10, 11]. Many researchers focus on the role of Bmi-1 in cell cycle regulation, stem cell self-renewal, and cell senescence [12]. It has also been demonstrated that the deficiency of Bmi-1 leads to significant mitochondrial dysfunction accompanied by increased and sustained high levels of ROS which is sufficient to induce senescence in organisms through the DNA damage response (DDR) pathway [12]. Bmi-1 is also highly expressed in skin tissue [13, 14]. Previous reports support a primary role of Bmi-1 in the maintenance of human keratinocyte survival [13, 15]. However, it is unclear whether Bmi-1 deficiency could lead to intrinsic skin aging by redox imbalance and DNA damage.

Pyrroloquinoline quinone (PQQ) is an aromatic tricyclic o-quinone which has been proven to be an antioxidant that protects living cells from oxidative damage in vivo and the biomolecules from artificially produced reaction oxygen species in vitro [16]. However, it is unclear whether PQQ can prevent Bmi-1 deficiency-induced skin aging by inhibiting oxidative stress and DNA damage. To answer this question, we used a Bmi-1^−/−^ mouse model in this study and fed Bmi-1^−/−^ mice with or without PQQ in the diet from 3 weeks old. We examined the changes in dermal morphology, proliferation, senescence, oxidative stress, and DNA damage to observe whether PQQ can prevent Bmi-1 deficiency-induced skin aging. This study will elucidate the role and mechanism of Bmi-1 in intrinsic skin aging and thus will provide a novel light for beauty.

## 2. Materials and Methods

### 2.1. Mice

The 1-, 9-, or 18-month-old WT mice were purchased from Vital River Laboratory Animal Technology Co. Ltd. (Beijing, China) and were divided into three groups, 7 mice per group. They were euthanized to harvest the skin and various organs for Bmi-1 expression level analysis.

Bmi1 heterozygous (Bmi1^+/-^) mice were kindly presented by professor Dengshun Miao. We crossed adult Bmi-1^+/-^ male mice with adult Bmi-1^+/-^ female mice to obtain Bmi-1 homozygous (Bmi-1^−/−^) mice and their WT littermates [11]: (1) seven WT littermates fed on a regular diet containing the basic nutrients needed for the growth of mice, provided by Beijing Cooperation Feed Co. Ltd., China; (2) seven Bmi-1^−/−^ mice fed on the regular diet; and (3) seven Bmi-1^−/−^ mice fed on the diet containing PQQ (4 mg kg^−1^ diet) started from 3 weeks old for 5 weeks. Purified PQQ was given free by Professor Chuanjun Wen in Academy of Life Science, Nanjing Normal University. PQQ-supplemented diet was made in Beijing Cooperation Feed Co. Ltd., China. All mice were sacrificed at 8 weeks of age. This study was carried out strictly in compliance with the guidelines of the Institutional Animal Care and Use Committee of Nanjing Medical University.

### 2.2. Histology

The skin was harvested for histologic processing as described [17]. The paraffin sections were stained for hematoxylin and eosin (H&E) or histochemically for total collagen, elastic fiber, and SA-*β*-gal as described and immunohistochemically as described below.

### 2.3. Aldehyde Red Staining

The aldehyde red staining kit (Solarbio) was used to detect the elastic fiber in paraffin sections. We performed strictly following the instructions of the manufacturer.

### 2.4. Immunohistochemistry

Immunohistochemical staining was performed following as described [17]. Primary antibodies against Bmi-1 antibody (Cell Signaling Technology), type I collagen (SouthernBiotech), PCNA (Santa Cruz), *γ*-H2AX (Cell Signaling Technology), 8-OHdG (Abcam), MMP1 (Proteintech), and MMP13(Abcam) were used.

### 2.5. Western Blot

Western blot was performed following as described [17]. Primary antibodies including p53 (Santa Cruz), p21 (Santa Cruz), p19 (Cell Signaling Technology), SOD2 (Cell Signaling Technology), *γ*-H2AX (Abcam), p-CHK2 (Abcam), MMP3 (Abmart), and *β*-actin (Abcam) were used.

### 2.6. Detection of ROS Levels

Total skin cells of 8-week-old mice were prepared as single-cell suspensions. Intracellular ROS analysis was performed as described [17].

### 2.7. Biochemistry Assay

The fresh skin was homogenized and centrifuged to obtain the supernatant for biochemical determination. The H_2_O_2_ and T-AOC levels in the skin were performed by using the commercial kits according to the manufacturer's instructions (Jiancheng Bioengineering).

### 2.8. Quantitative Real-Time RT-PCR

Total RNA was isolated from skin tissue by using TRIzol reagent (Invitrogen) according to the manufacturer's instructions. Skin mRNA levels were performed by RT-PCR or quantified by qRT-PCR as described. The primer sequences for mouse Bmi-1 were used: forward primer 5′-ATCCCCACTTAATGTGTGTCCT-3′ and reverse primer 5′-CTTGCTGGTCTCCAAGTAACG-3′.

### 2.9. Statistical Analysis

All data were performed using SPSS software (Version 19.0, SPSS Inc.). Measured values were expressed as mean ± SD and analyzed by Student's *t*-test. Comparisons were described using one-way ANOVA between groups. Qualitative value was performed as percentages and analyzed by a chi-square test as indicated. Statistically significance was determined when *P* < 0.05.

## 3. Results

### 3.1. The Expression of Bmi-1 in the Skin

**Figure 1:**
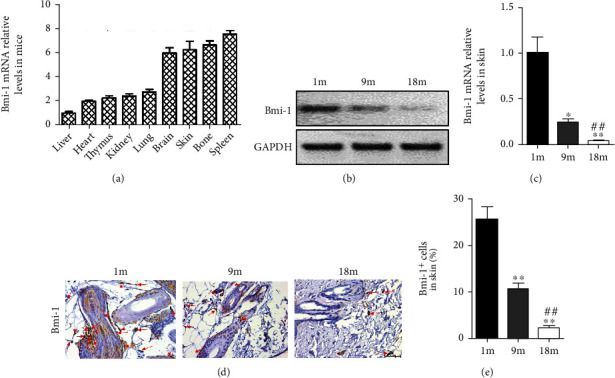
The expression of Bmi-1 in the skin. (a) qRT-PCR analysis of mRNA levels for Bmi-1 expression in organ extracts from 8-week-old mice. The expression level is calculated as a ratio to GAPDH, expressed relative to the liver. (b) Representative RT-PCR of skin extracts showing expression of Bmi-1; GAPDH was the loading control. (c) Bmi-1 mRNA relative levels detected by qRT-PCR, calculated as a ratio to GAPDH, expressed relative to WT. (d) Immunohistochemical analysis for expression of Bmi-1 in the skin from 1 m-, 9 m-, and 18 m-old mice. (e) The positive cell percentages of Bmi-1 were determined by image analysis. Values are shown as the mean ± SD of seven determinations per group. ^∗^*P* < 0.05 and ^∗∗^*P* < 0.01 versus 1 m-old mice; ^##^*P* < 0.01 versus 9 m old mice.

The Bmi-1 mRNA levels were detected in multiple organs from WT mice at 8 weeks old via real-time RT-PCR, including the liver, heart, thymus, kidney, lung, brain, skin, bone, and spleen. We found that, compared with other organs, Bmi-1 shows a relatively high expression level in the skin (Figure 1(a)). In order to observe the dynamic changes of Bmi-1 in the skin with age, Bmi-1 expression levels of the skin in different age groups of mice were examined by RT-PCR (Figures 1(b) and 1(c)) and immunohistochemistry (Figures 1(d) and 1(e)). We used mice in the following three age groups: 1-month-old mice (pubertal stage), 9-month-old mice (mature stage), and 18-month-old mice (early stages of aging) [18]. Our data revealed that Bmi-1 expression levels gradually decreased with age.

### 3.2. The Effect of PQQ on Skin Morphology of Bmi-1^−/−^ Mice

**Figure 2:**
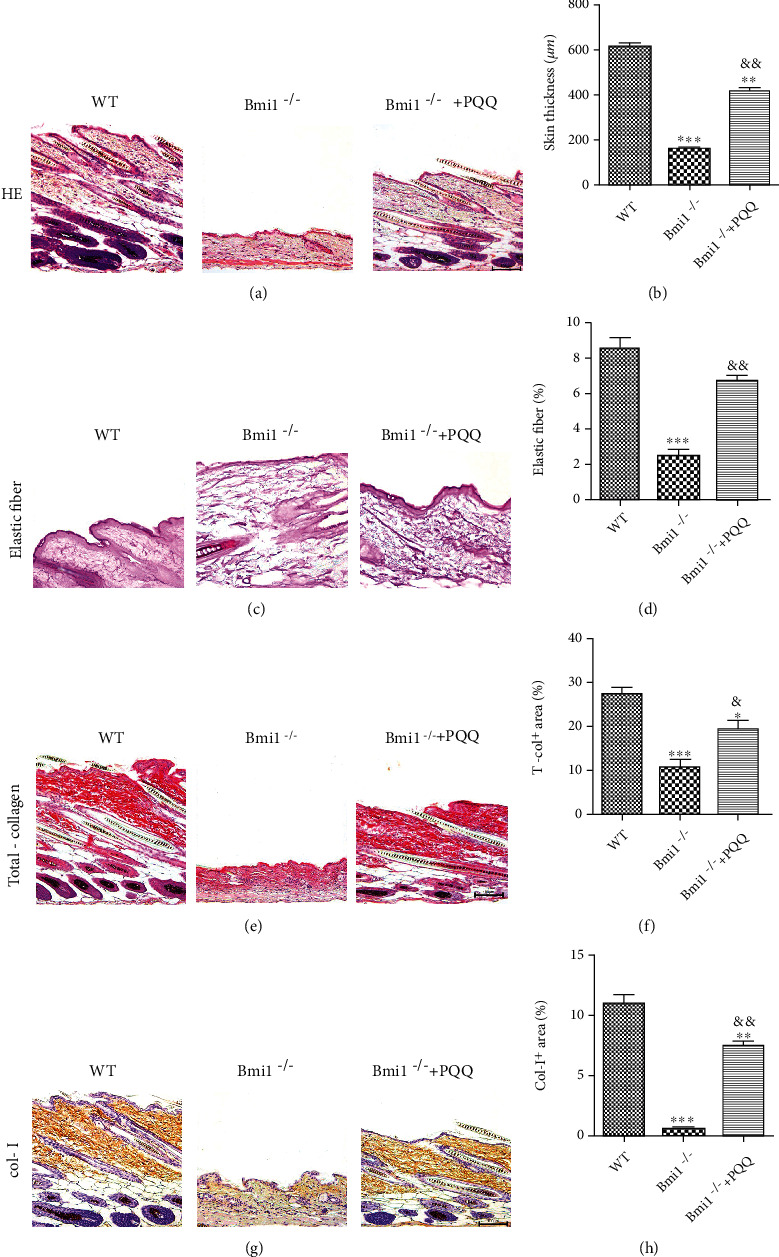
The effect of PQQ on skin morphology of Bmi-1^−/−^ mice. (a) Representative micrographs of paraffin embedded skin sections from WT, Bmi-1^−/−^, and Bmi-1^−/−^+PQQ mice stained for HE, (c) elastic fiber, (e) total collagen, and (g) immunohistochemically for type I collagen. (b) The skin thickness, the percentage of (d) elastic fiber-positive, (f) total collagen-positive, and (h) type I collagen-positive area were calculated and presented using image analysis. Values are shown as the mean ± SD of seven determinations per group. ^∗^*P* < 0.05, ^∗∗^*P* < 0.01, and ^∗∗∗^*P* < 0.001 versus WT mice; ^&^*P* < 0.05 and ^&&^*P* < 0.01 versus Bmi-1^−/−^ mice.

To observe the effect of Bmi-1 deficiency on the skin, we detected the alterations in skin morphology at 8 weeks of age. Compared with WT littermates, the alterations of skin histological pattern in Bmi-1^−/−^ mice were changed significantly. The dorsal skin of Bmi-1^−/−^ mice showed decreased vertical thickness of skin, sparse hair follicles (Figures 2(a) and 2(b)), and thinner and more irregular elastic fibers (Figures 2(c) and 2(d)). And the positive area and density of total collagen fibers (Figures 2(e) and 2(f)) and type І collagen (Figures 2(g) and 2(h)) were decreased obviously in Bmi-1^−/−^ mice compared with WT mice. These results suggested that Bmi-1 deficiency can lead to skin aging. However, PQQ markedly rescued the skin aging morphology of Bmi-1^−/−^ mice.

### 3.3. The Effect of PQQ on Cell Proliferation and Senescence in Skin of Bmi-1^−/−^ Mice

**Figure 3:**
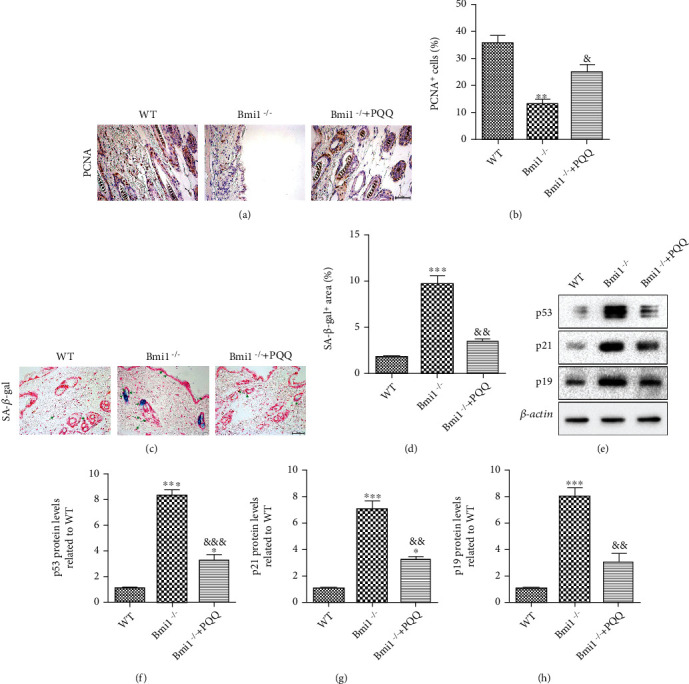
The effect of PQQ on cell proliferation and senescence in the skin of Bmi-1^−/−^ mice. Representative micrographs of paraffin-embedded skin sections from 8-week-old WT, Bmi-1^−/−^, and Bmi-1^−/−^+PQQ mice stained for (a) PCNA and (c) SA-*β*-gal. The percentage of (b) PCNA-positive and (d) SA-*β*-gal-positive cells was calculated and presented using image analysis. (e) Western blot analysis of protein level for p53, p21, and p19 in the skin from the WT, Bmi-1^−/−^, and Bmi-1^−/−^+PQQ mice. *β*-Actin was used as a loading control. (f) p53, (g) p21, and (h) p19 protein levels relative to *β*-actin protein levels were determined using densitometric analysis and expressed relative to WT. Values are shown as the mean ± SD of seven determinations per group. ^∗^*P* < 0.05, ^∗∗^*P* < 0.01, and ^∗∗∗^*P* < 0.001 versus WT mice; ^&^*P* < 0.05, ^&&^*P* < 0.01, and ^&&&^*P* < 0.001 versus Bmi-1^−/−^ mice.

To further evaluate whether the effect of PQQ on the skin aging phenotypes of Bmi-1^−/−^ mice was related to the alterations of skin cell proliferation and cell senescence, the marker of cellular proliferation, PCNA, and the markers of cellular senescence, senescence-associated *β*-galactosidase (SA-*β*-gal), p53, p21, and p19 protein levels, were examined by immunohistochemical staining, histochemical staining, or Western blots. We found that, compared with WT littermates, the percentage of PCNA-positive cells (Figures 3(a) and 3(b)) in the skin of Bmi-1^−/−^ mice was decreased, while the percentage of SA-*β*-gal-positive cells (Figures 3(c) and 3(d)) and the protein expression levels of p53, p21, and p19 (Figures 3(e) and 3(h)) were increased clearly. However, these alterations were rescued markedly in Bmi-1^–/–^ mice supplemented with PQQ. These results indicated that the effect of PQQ on the skin aging phenotypes of Bmi-1^−/−^ mice may be associated with increasing cell proliferation and inhibiting cell senescence in skin.

### 3.4. The Effect of PQQ on Redox Balance in the Skin of Bmi-1^−/−^ Mice

**Figure 4:**
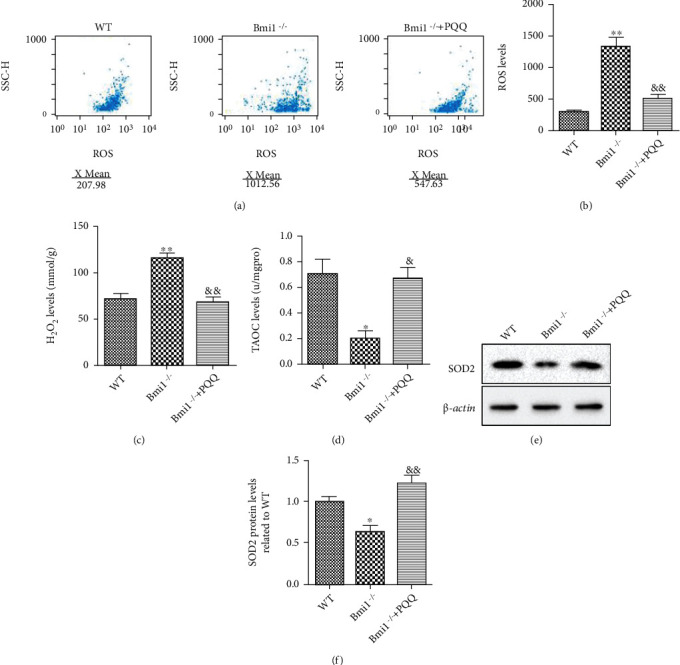
The effect of PQQ on redox balance in the skin of Bmi-1^−/−^ mice. (a) Cellular ROS levels in the skin from 8-week-old WT, Bmi-1^−/−^, and Bmi-1^−/−^+PQQ mice were analyzed by flow cytometry. (b) ROS levels were calculated. Biochemistry analysis of skin extracts from 8-week-old WT, Bmi-1^−/−^, and Bmi-1^−/−^+PQQ mice for (c) H_2_O_2_ levels or (d) TAOC. (e) Western blot analysis of protein level for SOD2 in the skin from the WT, Bmi-1^−/−^, and Bmi-1^−/−^+PQQ mice. *β*-Actin was used as a loading control. (f) SOD2 protein levels relative to *β*-actin protein levels were determined using densitometric analysis and expressed relative to WT. Values are shown as the mean ± SD of seven determinations per group. ^∗^*P* < 0.05 and ^∗∗^*P* < 0.01 versus WT mice; ^&^*P* < 0.05 and ^&&^*P* < 0.01 versus Bmi-1^−/−^ mice.

Based on the observations that oxidative stress in the skin is one of the core mechanisms mediating skin aging, we further determined whether skin aging phenotypes in Bmi-1^−/−^ mice were associated with an alteration of redox balance. The oxidative stress marker, cellular ROS, H_2_O_2_, the total antioxidant capacity (TAOC), and SOD2 protein expression level in the skin were measured. The results showed that ROS (Figures 4(a) and 4(b)) and H_2_O_2_ (Figure 4(c)) levels in the skin were increased obviously in Bmi-1^−/−^ mice compared with WT littermates. To further determine whether an evident increase of oxidative stress induced by Bmi-1 deficiency in the skin was associated with reduced antioxidant capacity, the total antioxidant capacity and the SOD2 protein expression level were detected in the skin. The results showed that Bmi-1^−/−^ mice exhibited a significantly lower TAOC (Figure 4(d)) and the SOD2 protein expression level (Figures 4(e) and 4(f)) than WT mice. Our data demonstrated that PQQ could clearly increase antioxidant capacity of Bmi-1^−/−^ mice. These results indicated that PQQ may increase cell proliferation and inhibit cell senescence in skin of Bmi-1^−/−^ mice by inhibiting oxidative stress.

### 3.5. The Effect of PQQ on DNA Damage in the Skin of Bmi-1^−/−^ Mice

**Figure 5:**
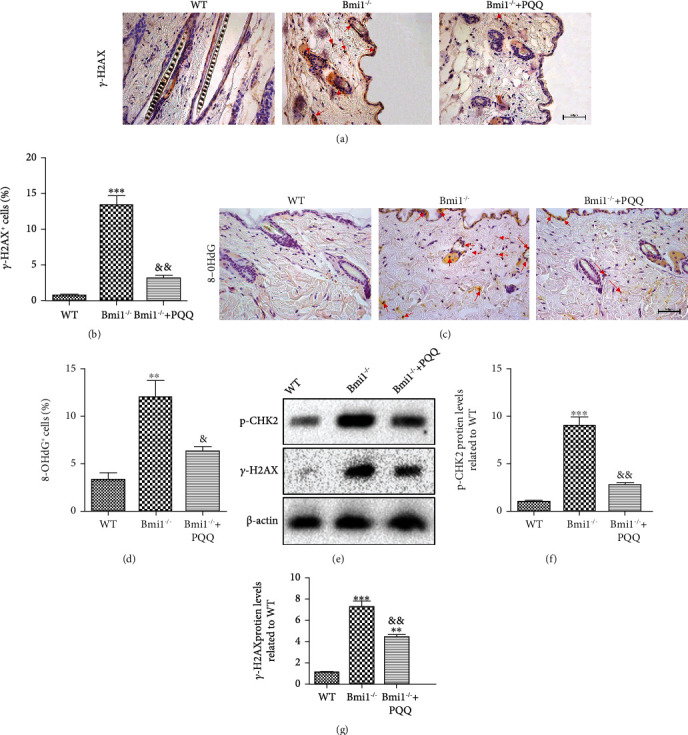
The effect of PQQ on DNA damage in the skin of Bmi-1^−/−^ mice. Representative micrographs of paraffin-embedded skin sections from 8-week-old WT, Bmi-1^−/−^, and Bmi-1^−/−^+PQQ mice stained immunohistochemically for (a) *γ*-H2AX and (c) 8-OHdG. The percentage of (b) *γ*-H2AX-positive and (d) 8-OHdG-positive cells was calculated and presented using image analysis. (e) Western blot analysis of protein level for p-CHK2 and *γ*-H2AX in the skin from the WT, Bmi-1^−/−^, and Bmi-1^−/−^+PQQ mice. *β*-Actin was used as a loading control. (f) p-CHK2 and (g) *γ*-H2AX protein levels relative to *β*-actin protein levels were determined using densitometric analysis and expressed relative to WT. Values are shown as the mean ± SD of seven determinations per group. ^∗∗^*P* < 0.01 and ^∗∗∗^*P* < 0.001 versus WT mice; ^&^*P* < 0.05 and ^&&^*P* < 0.01 versus Bmi-1^−/−^ mice.

The activation of the DNA damage response can be triggered by oxidative stress. Therefore, to determine whether the increase of oxidative stress in Bmi-1^−/−^ mice can induce DNA damage, the DNA damage markers, *γ*-H2AX, 8-OHdG, and p-CHK2, were determined by immunohistochemical staining or Western blots in the skin. The results revealed that the percentage of *γ*-H2AX -positive cells (Figures 5(a) and 5(b)) and 8-OHdG-positive cells (Figures 5(c) and 5(d)) and the protein expression levels of p-CHK2 (Figures 5(e) and 5(f)) and *γ*-H2AX (Figures 5(e) and 5(g)) in the skin of Bmi-1^−/−^ mice were increased dramatically compared with WT littermates. These results indicated that elevated oxidative stress in the skin induced by Bmi-1 deficiency could induce DNA damage. However, compared with Bmi-1^−/−^ mice fed with the regular diet, PQQ-supplemented Bmi-1^−/−^ mice showed a marked decrease in DNA damage. Our results indicated that PQQ supplementation could inhibit DNA damage triggered by oxidative stress in Bmi-1^−/−^ mice.

### 3.6. The Effect of PQQ on MMPs in the Skin of Bmi-1^−/−^ Mice

**Figure 6:**
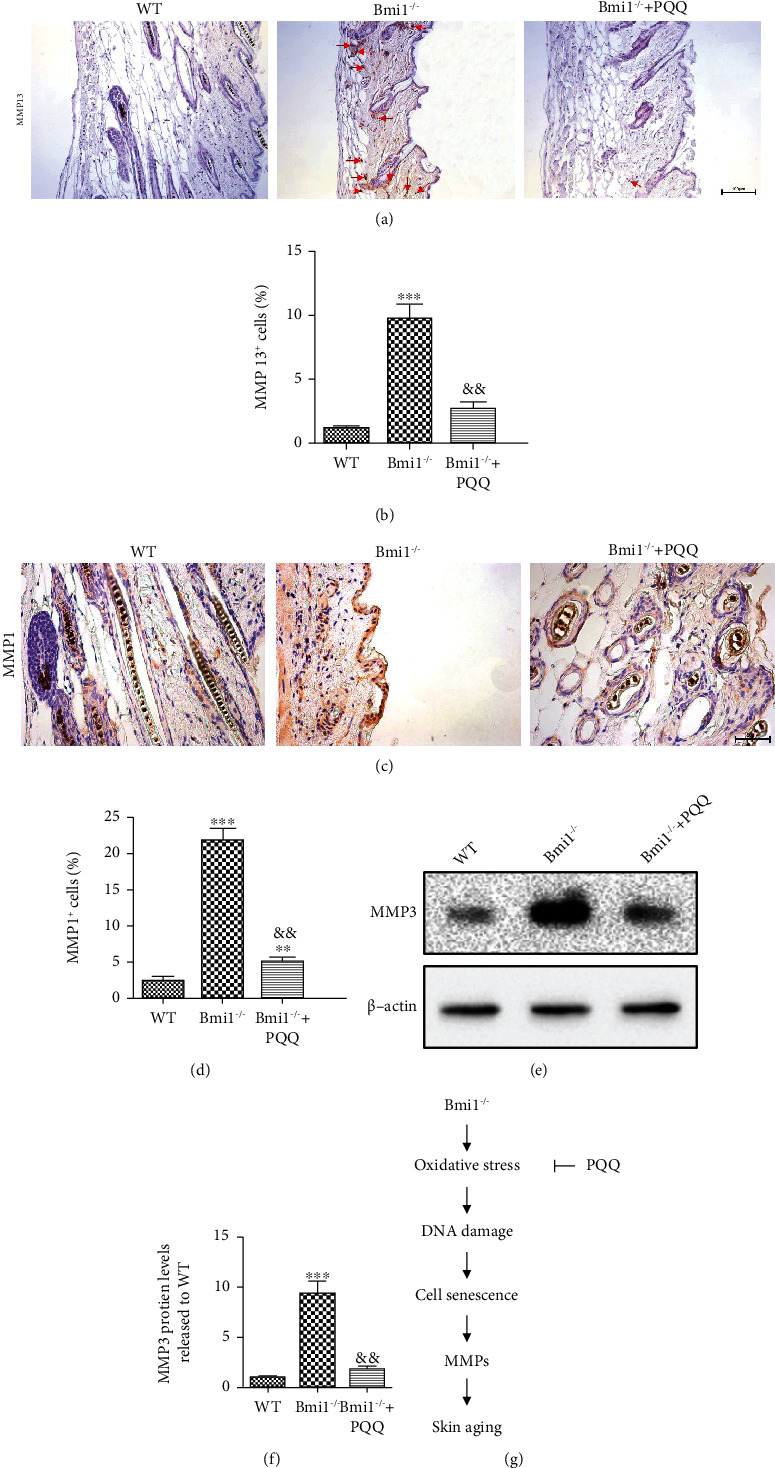
The effect of PQQ on MMPs in the skin of Bmi-1^−/−^ mice. Representative micrographs of paraffin-embedded skin sections from 8-week-old WT, Bmi-1^−/−^, and Bmi-1^−/−^+PQQ mice stained immunohistochemically for (a) MMP-13 and (c) MMP-1. The percentage of (b) MMP-13-positive and (d) MMP-1-positive cells was calculated and presented using image analysis. (e) Western blot analysis of protein level for MMP-3 in the skin from the WT, Bmi-1^−/−^, and Bmi-1^−/−^+PQQ mice. *β*-Actin was used as a loading control. (f) MMP-3 protein levels relative to *β*-actin protein levels were determined using densitometric analysis and expressed relative to WT. (g) Schematic diagram of PQQ prevents Bmi-1 deficiency-induced skin aging by inhibiting oxidative stress and DNA damage. Values are shown as the mean ± SD of seven determinations per group. ^∗∗^*P* < 0.01 and ^∗∗∗^*P* < 0.001 versus WT mice; ^&&^*P* < 0.01 versus Bmi-1^−/−^ mice.

Activation of DNA damage response can result in cell senescence which can trigger the secretion of MMPs. To determine whether activated DNA damage in the skin of Bmi-1^−/−^ mice could lead to the increase of MMPs, we detected the expression levels of MMP-1, MMP-3, and MMP-13 by immunohistochemical staining or Western blots. We found that the percentage of MMP-13-positive cells (Figures 6(a) and 6(b)) and MMP-1-positive cells (Figures 6(c) and 6(d)) and the protein expression levels of MMP-3 (Figures 6(e) and 6(f)) in the skin of Bmi-1^−/−^ mice were significantly increased compared with WT littermates. However, PQQ supplementation inhibited obviously MMP expression levels in the skin. The above data indicated that PQQ could partially inhibit MMPs in the skin of Bmi-1^−/−^ mice.

## 4. Discussion

The polycomb gene Bmi-1 is necessary for the maintenance of stem cells and rapid cell division [19]. The role of Bmi-1 in stem cell has been widely reported. Recent studies suggested that Bmi-1 also regulates cell cycle, controls mitochondrial function, and regulates oxidative stress [20–22]. However, the function and mechanism of Bmi-1 in the skin remain unclear. In this study, we first demonstrated that the gene expression level of Bmi-1 in the skin is higher than other organs and gradually decreased with age. We also proved that Bmi-1 deficiency leads to skin aging with increased oxidative stress and DNA damage, decreased cell proliferation, increased cell senescence and MMPs expression. These findings provide evidence that Bmi-1 plays a protective role against intrinsic skin aging.

Consist with previous studies [14, 23], in our study, we confirmed that hair follicles, keratinocytes, and fibroblasts have relatively stronger expression levels of Bmi-1 localization. Long considered both physiologic and inevitable, skin aging is a degenerative phenomenon progressively with age [24]. In order to observe the correlation between Bmi-1 and age, we tracked the dynamic changes of Bmi-1 expression levels in the skin from 1-, 9-, and 18-month-old mice. We found that there was an age-related decline for the expression level of Bmi-1 in the skin. These findings revealed that the downregulation of Bmi-1 expression levels in the skin with age may be associated with skin aging.

There are three layers in the skin: the epidermis, dermis, and subcutaneous tissue. As skin ages, the three layers will undergo degenerative alterations, especially in the dermis [25]. One of the major mechanisms of skin aging is thought to be the reduction of extracellular matrix, especially the amount of collagen in the dermis. In aged skin, the collagen production decreases and its degradation increases, thus resulting in an overall decrease in the amount of collagen. Most antiaging strategies target to reverse this process [26, 27]. In our reports, we found that Bmi-1^−/−^ mice showed an internal aging phenotype, which was manifested by decreased vertical thickness of the skin, sparse hair follicles, fragmented and coarsely distributed collagen fiber bundles, and decreased collagen amount compared with WT mice. Senescent cells have been shown to accumulate in chronologically aging skin and may result in age-related skin changes and lesions [28]. We further confirmed the increase of cell senescence in the skin of Bmi-1^−/−^ mice, including significant increases in SA-*β*-gal-positive cells.

Aging is a complex biological process which is a major factor in the development of many age-related diseases. Antiaging mechanisms, including antioxidation, DNA damage repair, maintenance of protein homeostasis, and inhibition of cell senescence, were widely studied [29, 30]. It is reported that the redox imbalance caused by the accumulation of reactive oxygen species (ROS) can cause damage to lipids, proteins, nucleic acids, and organelles, thus leading to the occurrence of cell senescence, which is one of the core mechanisms that mediates skin aging [2]. Studies have shown that antioxidants can offset H_2_O_2_-mediated lipid peroxidation and DNA oxidative damage in skin cells, and the reduction in collagen synthesis is associated with oxidative stress [31, 32]. In our current study, we found that elevated oxidative stress in the skin of Bmi-1^−/−^ mice, including a dramatical increase in the levels of intracellular ROS and H_2_O_2_, resulted from decreased endogenous antioxidant levels, including significantly downregulated T-AOC activities and SOD2 protein level. It is thought that in human skin, ROS can lead to further oxidative damage and genomic instability to DNA [33, 34]. Consistent with other studies, in which different cells derived from Bmi-1 deficiency mice have an increased production of the intracellular ROS levels and subsequent DDR involvement [11, 12, 35, 36], our results confirmed that the DDR occurred in the skin of Bmi-1^−/−^ mice, including marked increases in *γ*-H2AX, 8-OHdG, and p-CHK2. DNA damage response can drive cells into senescence which can cause the secretion of numerous biologically active factors, including MMPs. MMPs are major collagenolytic enzymes that hydrolyze collagen protein and result in hydrolysis, destruction, and restructuring of extracellular matrix [25]. Our data showed obvious increases in MMP-1, MMP-3, and MMP-13 which may contribute to skin aging of Bmi-1^−/−^ mice by enhancing collagen degradation.

PQQ actions as an accessory cofactor in mitochondriogenesis and enhances NAD+-dependent sirtuin activity and the expression of sirtuin targets [37]. In the past decade, a large amount of evidence has shown that PQQ is a strong antioxidant, including modulating mitochondrial quantity and function in aged rats and ameliorating oxidative stress and lipid peroxidation in the brain [37, 38]. PQQ also can catalyze the nonenzymatic oxidation of the epsilon amine function of lysyl groups to aldehydic functions in proteins, such as elastin and collagen [39]. Mice fed without PQQ reduced the lysyl oxidase in the skin by 70-90%, leading to the dissolution of collagen and the dry skin [37]. In human study, PQQ supplementation significantly improves the skin condition of female subjects with dry skin [40]. In vitro experiments found that PQQ had protective effects on UVA irradiation-induced human dermal fibroblast aging [41]. And in Bmi-1^−/−^ mice, studies have revealed that PQQ could protect mitochondrial activity of other tissues from oxidative damage, such as the liver [42], mandible [43], long bone [44], and kidney [11]. In our current study, we found oral intake of PQQ largely reduced oxidative stress, DNA damage, cell senescence, and MMPs levels in the skin of Bmi-1^−/−^ mice. These results were consistent with previous studies which suggested that PQQ participates in the antioxidant mechanism in skin aging of Bmi-1^−/−^ mice.

Taken all together, our conclusion was drawn that PQQ could suppress Bmi-1 deficiency-induced skin aging by decreasing oxidative stress and DNA damage, promoting cell proliferation, inhibiting cell senescence and MMP secretion, then increasing collagen syntheses, and reducing collagen degradation (Figure 6(g)). The present study indicated for the first time that Bmi-1 and PQQ may play a key role in protection from skin aging. Our findings implied that Bmi-1 may be a novel target for delaying skin aging, which provides a theoretical and experimental basis for the clinical application of PQQ in the prevention and treatment of skin aging.

## Data Availability

The data used to support the findings of this study are included within the article.
